# Should COVID-19 Mother Breastfeed her Newborn Child? A Literature Review on the Safety of Breastfeeding for Pregnant Women with COVID-19

**DOI:** 10.1007/s13668-020-00343-z

**Published:** 2021-01-04

**Authors:** Harshil Bhatt

**Affiliations:** 1 Goshen Hospital, Goshen, IN USA; 2 Indiana University School of Medicine, South Bend, IN USA

**Keywords:** COVID-19, Breastfeeding, Breast milk, Pregnancy, SARS-CoV-2, Infant, Newborn

## Abstract

**Purpose of Review:**

Breastfeeding is beneficial to both the newborn and the mother. During the COVID-19 pandemic, concerns have been raised on whether the SARS-CoV-2 virus could be transmitted from COVID-19 positive mother to the newborn through breastmilk. The purpose of this review is to examine the available evidence on the risks of transmission of infection from COVID-19 mothers to their newborns through breastfeeding.

**Recent Findings:**

Data is very limited in this regard, with only a few smaller case series, and case reports have been published so far. In most of the studies, breastmilk samples from COVID-19 mothers tested negative for the virus. In the case reports where the virus was detected in breastmilk and the infants were diagnosed with COVID-19, it remained unclear whether the disease was transmitted through breastmilk or direct contact or through delivery. Another hypothesis is that the viral antibodies could pass to the newborn passively through breastmilk of COVID-19 positive mothers and give immunity to the child, but data is minimal.

**Summary:**

Based on the currently available limited evidence and recognizing the benefits of breastfeeding, it may be concluded that if the health of the mother and her newborn allows, direct breastfeeding or extracted breastmilk should be encouraged by the healthcare providers after a careful discussion of the risks of vertical transmission to the mother and her family. Preventive measures should be taken by COVID-19 mothers to prevent droplet transmission of infection to the infants while breastfeeding.

## Introduction

Coronavirus disease-2019 (COVID-19), declared a pandemic on March 11, 2020, by the World Health Organization (WHO), started as an outbreak of pneumonia in Wuhan, China, in December 2019. Later, it was discovered that the disease was caused by a single-stranded RNA virus, which was named SARS-CoV-2 (severe acute respiratory syndrome coronavirus-2). The virus is known to be transmitted by droplets of an infected individual and leads to a variety of symptoms affecting respiratory and other systems in the body [1]. COVID-19 has been spreading globally very rapidly. As of October 25, 2020, over 42 million confirmed cases were reported worldwide [2].

Pregnant women and newborns can be considered a high-risk population during the COVID-19 pandemic because their vulnerability to acquire any infection is higher in general due to lowered immunity during this period. Even though initial data from smaller studies have suggested that the pregnancy does not increase the risk of getting COVID-19 infection and the severity of illness is not different from the general population, caution still must be exercised by both the pregnant women and their clinicians as there is a lot that we are not aware of this disease [3].

Breastfeeding is beneficial to both the mother and the baby. Breastmilk can serve as the source of many required nutrients to the breastfed newborns during the first few months of life and provide the much-needed immunity as well. Breastfeeding protects the mother from the development of breast and ovarian cancer and helps her recover during the postpartum period [4]. WHO has also recommended breastfeeding exclusively for the first 6 months and then continuing it up to the age of 2 years and beyond [5].

The following represents a thorough review of the literature, including the research and recommendations published so far related to breastfeeding during the COVID-19 pandemic. The comprehensive review focuses on the available evidence on the transmission of SARS-CoV-2 through breastmilk and recommendations for nursing mothers who are COVID positive on safe and protective breastfeeding.

## Discussion

### Detection of SARS-CoV-2 in Breastmilk

The biggest fear of breastfeeding newborns during the COVID-19 period is the fear of transmitting the virus through breastmilk. There is still not a substantiate amount of evidence available regarding the transmission of SARS-CoV-2 in breastmilk. Much of our knowledge comes from the data obtained from smaller studies and case reports (Table 1) [6–17]. To date, there have been no large-scale cohort studies or case-control studies evaluating the transmission of COVID-19 through breastmilk.

**Table 1:** Studies examining the transmission of SARS-CoV-2 in breastmilk

Study	Number of positive mothers	Breastmilk samples tested with SARS-CoV-2-RT-PCR	Results of breastmilk samples
Liu et al. [6] (January 31 to February 29, 2020)	10 (laboratory confirmed positive) 9 (clinically diagnosed)	10	10 negative
Groß R et al. [7•]	2	Mother I—4 Mother II—7	Mother I—all negative Mother II—4 out of 7 positive (newborn unaffected)
Wu et al. [8] (January 31 to March 9, 2020)	5	3	1 positive (newborn positive but the mode of transmission unclear) 2 negative
Kirtsman et al. [9]	1	1	1 positive (newborn positive)
Chen et al. [10] (January 20 – January 31, 2020)	9	6	6 negative (newborns negative)
Zhu et al. [11] (January 20 – February 5, 2020)	9	9	9 negative (all tested 9 newborns negative)
Li et al. [12]	1	1	Negative (newborn was not tested)
Chambers et al. [13] (March 27 – May 6, 2020)	18	64	1 sample positive, but the virus was not replication-competent (the newborn related to the positive breast milk sample was not tested)
Tam et al. [14]	1	7	1st and 7th samples positive (10 days apart) The infant (8 months) was positive. The contamination of the sample was not ruled out.
Dong et al. [15]	1	1	Negative (day 6th after delivery) (newborn negative)
Marín Gabriel MÁ et al. [16]	7	7 (colostrum samples 1-h post-delivery)	All negative
Salvatori et al. [17]	2	2	Both negative (newborns were positive, but horizontal transmission was suspected)

*RT-PCR*, reverse transcription-polymerase chain reaction

Furthermore, the scientific brief on breastfeeding and COVID-19 published by WHO on June 23, 2020, included a living systemic review performed by Centeno-Tablante et al., which showed that out of 46 COVID-19 positive mothers whose breastmilk samples were tested for SARS-CoV-2, 43 breastmilk samples tested negative, and only three samples tested positive. Out of the three mothers whose breastmilk samples were positive, only one infant tested positive for the virus, but the route of infection (breastmilk or close contact) could not be determined [18••, 19]. In another systemic review by Elshafeey et al., all 26 breastmilk samples from 26 mothers were negative for COVID-19 [20]. A rapid systemic review of eight studies by Martins-Filho PR et al. showed that breastmilk samples of 24 pregnant women diagnosed with SARS-CoV-2 by PCR test were negative for the virus [21].

However, in addition to being smaller studies, there are several limitations associated with these reported studies. In most of the studies where the virus was detected in breastmilk, no precise information was available on sample collecting techniques. Also, only few of these studies tested breastmilk samples for the virus for several consecutive days. Groß R et al. reported that the breastmilk samples from one of the mothers in the study tested positive for four consecutive days before it tested negative [7•]. Chambers et al. reported that one out of 64 breastmilk samples was positive for the virus, but the same sample had tested negative 2 days prior as well as 12 and 41 days later [13]. Tam et al. also reported the virus’s detection in the breast milk samples of a woman on day 1 and day 10 with five negative samples in between [14]. But other studies did not perform such analysis of breastmilk samples for successive days. It means that even if the virus is excreted through the breastmilk, there is no clear data on the duration (in days) that the virus can be found in breastmilk. Besides, when the newborns test positive for COVID-19 and whose mothers are positive at the time of birth, it becomes challenging to establish whether the source or route of transmission is through breastmilk, through droplet transmission through close contact, transplacental, or even through the passage of the newborn through the birth canal. Hence, it becomes imperative to conduct more studies to look for possible transmission of the virus through breastmilk.

### Detection of Viral Antibodies in Breastmilk

It is well-known that breastmilk protects the infant by providing antibodies during the first few months of life while the infant’s immune system is still developing. Whether the same is true in the case of COVID-19 is still not entirely known. There have been few reports suggesting the presence of IgG and IgA (immunoglobulins G and A) antibodies in breastmilk, which could offer immunity to the newborn from COVID-19 [22, 23]. Another preprint article revealed that the secretory IgA was present in 12 of 15 breastmilk samples from COVID-19 positive mothers [24]. Even though this data is encouraging, detailed studies evaluating the protective effect of these antibodies against COVID-19 are warranted.

### Recommendations for Breastfeeding for COVID-19 Mothers

During the earlier months of COVID-19, there appeared to be a lack of consensus regarding breastfeeding for COVID-19 positive mothers. In February 2020, Chinese experts recommended against breastfeeding for mothers with suspected or confirmed COVID-19. They advised initiating breastfeeding only if the mother and her breast milk samples tested negative [25].

Based on the currently available evidence, WHO has recommended that the mothers with confirmed or suspected COVID-19 should continue to breastfeed since the benefits of breastfeeding to both the mothers and newborns significantly outweigh the risk of transmission of COVID-19 to the newborns [26]. These guidelines echo with the ones from the Centers for Disease Control and Prevention (CDC) and the Royal College of Obstetricians and Gynecologists. The mother should be willing to follow all necessary preventive measures to avoid the spread of infection to the newborn since infection can still pass from the mother to the baby through droplets. When the mothers must stay separated from the newborn due to their clinical condition or those mothers who are not comfortable following the preventive measures or who do not feel safe to breastfeed their newborns directly, the option of expressed breastmilk should be recommended (Table 2) [27]. There is no clear evidence currently to support measures such as cleaning the breast before breastfeeding or disinfecting bottles or bags (accessories for collecting milk) before use as ways to decrease transmission of the virus [28].

**Table 2:** Preventive measures for safe breastfeeding for COVID-19 mothers

• Wash hands with soap and water before breastfeeding or extracting breastmilk • Use hand sanitizer with at least 60% alcohol if soap and water are unavailable • Wear a face mask to cover mouth and nose during breastfeeding or extracting breastmilk • Properly clean and sanitize breast pumps before using them according to the manufacturer’s guidelines • Expressed breastmilk should be fed to the newborn by a healthy caregiver	

## Conclusion

After examining the limited data published so far, we can conclude that our knowledge of the transmission of the SARS-CoV-2 virus through breastmilk is quite insufficient. At the same time, we cannot ignore the evidence that proves the benefits of breastfeeding to both the mother and the newborn. Even though, based on the limited set of literature, it can be inferred that if the health of both the mother and her newborn permits, continuation of breastfeeding should be advocated to the mothers with COVID-19; healthcare providers should still cautiously discuss the risks of vertical transmission with the patients to help them make a well-informed decision. Providers should also educate the mothers about the disease process as well as the precautions required to prevent contact transmission of COVID-19 to the newborns. When direct breastfeeding is not feasible due to health concerns, giving extracted breastmilk to the newborns can be considered. An in-depth analysis of virus pathophysiology and its transmission through breastmilk will be possible once more extensive studies are conducted to examine this correlation.

## Data Availability

The authors declare that data supporting the findings of this study are available within the article.

## Abbreviations

COVID-19: Coronavirus disease-2019
WHO: World Health Organization
SARS-CoV-2: Severe acute respiratory syndrome coronavirus-2
RT-PCR: Reverse transcription-polymerase chain reaction
IgG, IgA: Immunoglobulin G and A
CDC: Centers for Disease Control and Prevention
